# Skull-femoral traction and vertebral osteotomies for severe rigid scoliosis with trunk imbalance

**DOI:** 10.1097/MD.0000000000044134

**Published:** 2025-08-29

**Authors:** Yingliang Liu, Changlei Xu, Yingsong Wang, Zhi Zhao, Jingming Xie, Ni Bi, Jie Xiao, Xiaobing Tian

**Affiliations:** a Department of Orthopaedics, The Second Affiliated Hospital of Kunming Medical University, Kunming, Yunnan, China; b Department of Orthopaedics, The People’s Hospital of Chuxiong Yi Autonomous Prefecture, Chuxiong, Yunnan, China; c Department of Orthopaedics, The People’s Hospital of Wenshan Prefecture, Wenshan, Yunnan, China.

**Keywords:** posterior vertebral column resection, severe rigid scoliosis with trunk imbalance, skull-femoral traction, vertebral osteotomies

## Abstract

This study aimed to explore skull-femoral traction and posterior vertebral column resection (PVCR) for the treatment of severe rigid scoliosis with trunk imbalance. The study also aimed to compare the procedure to the non-traction procedure with matched analysis. From January 2007 to December 2021, 59 patients (traction group) with severe rigid scoliosis and trunk imbalance underwent skull-femoral traction and PVCR. For comparison, another 1:1 matched group of 59 patients (non-traction group) was also retrospectively reviewed. These patients were treated with PVCR alone. The spinal function was assessed using the Scoliosis Research Society-Questionnaire. Differences were considered statistically significant at *P* < .05. The age of the traction and non-traction groups were 21.1 ± 8.2 years and 21.4 ± 10.3 years, respectively (*P* > .05). The duration of skull-femoral traction was 24 days (range, 14–39 days). The operative time was 521 ± 101 and 679 ± 443 minutes, respectively (*P* < .05). The amount of intraoperative blood loss was 987 ± 446 and 5961 ± 3214 mL, respectively (*P* < .05). The number of resected vertebrae was 0.4 ± 0.3 vs 1.1 ± 0.6 (*P* < .05). The groups were followed up for 34.9 ± 7.3 months and 31.1 ± 6.6 months, respectively (*P* > .05). The total Scoliosis Research Society-22 Questionnaire Scores were 4.7 (range, 3.8–5) and 4.5 (range, 3.3–5), respectively (*P* > .05). In the treatment of severe rigid scoliosis with trunk imbalance, preoperative skull-femoral traction improves curve flexibility of the spine, decreasing osteotomy grade, PVCR, average number of 3-column vertebrae resection, operative time, and intraoperative blood loss. However, both traction and non-traction techniques achieve similar spine correction and functional outcomes with similar complications and modalities.

## 1. Introductions

Severe rigid scoliosis with trunk imbalance (SRSTI) is a group of spinal deformities (Cobb angle of the main curve > 90°, flexibility < 20%) and trunk imbalance.^[1]^ Daubs et al^[2]^ reported that 12% of adult scoliosis patients demonstrated trunk imbalance. The treatments are challenging due to a stiff deformity, prolonged surgical duration, massive blood loss, and potential neurologic deficit. Currently, the optimal traction protocol and approach remain debated.^[3]^

Nemani^[4]^ suggested a combination of anterior and posterior release and osteotomies but required a longer hospital stay and chest-related complications. Bradford and Tribus^[5]^ performed a series of anterior and posterior vertebral column resections (PVCRs), but the incidence of neurological deficits was as high as 17%. Halo-gravity traction is associated with minor postoperative complications but provides low traction strength and efficiency, especially for rigid and severe deformities in elderly or adult patients.^[6–10]^ Skull-femoral traction offers more powerful traction from both caudal and cephalic ends.^[11]^ It effectively mitigates the neurological risks of PVCR for extremely severe rigid and spinal curvature deformities.^[12]^ Skull-femoral traction can achieve maximum distance within 2 weeks and decreases the complication rate.^[13]^ Currently, the efficacy and matched analysis of craniofemoral traction for SRSTI are not reported in the literature.

This retrospective study aimed to evaluate the efficiency of preoperative skull-femoral traction and PVCR for the treatment of SRSTI. For comparison, we also included another 1:1 matched series of SRSTI treated with non-traction PVCR.

## 2. Materials and methods

The Ethical institutional review boards of the participating hospitals approved the study (No. PJ-2024-29). Informed consent was obtained from each patient or their parents.

**Figure 1.**
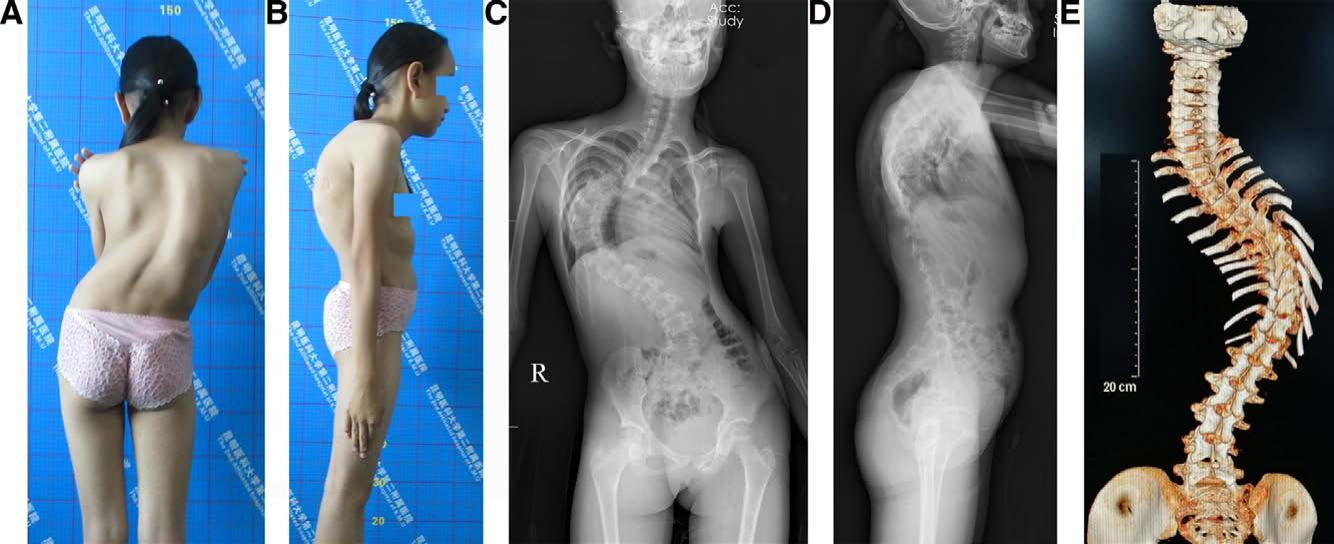
An 11-year-old female patient suffers from severe rigid scoliosis with trunk imbalance. (A) Preoperative posterior view of deformity. (B) Lateral view. (C) A preoperative anteroposterior (AP) X-ray shows a Cobb angle of 112°, radiographic shoulder height (RSH) of 3.4 cm, C7-central sacral vertical line (C7-CSVL) of 2.7 cm. (D) On a lateral X-ray, the Cobb angle is 80°, and the sagittal vertical axis (SVA) is 2.7 cm. (E) Preoperative 3-dimensional CT image reconstruction showing a posterior view of the spinal deformity. AP = anteroposterior, C7-CSVL = C7-central sacral vertical line, CT = computed tomography, RSH = radiographic shoulder height, SVA = sagittal vertical axis.

From January 2007 to December 2021, patients with SRSTI were treated with skull-femoral traction and vertebral osteotomies. Preoperative X-rays and computed tomography scans were obtained in all patients (Fig. 1A, B). The inclusion criteria were an established diagnosis of severe rigid scoliosis with <20% of correction on bending films and major curve >90°; trunk imbalance, C7-CSVL > 2 cm or SVA > 2.5 cm or RSH > 2cm; patients undergoing vertebral osteotomies; and idiopathic scoliosis; patients with a complete history, medical documents, and follow-ups. The exclusion criteria included a history of spine surgery; leg length discrepancy; patients undergoing other traction methods; unstable medical conditions such as heart failure or type II respiratory failure; and an active infection.

For comparison, we retrospectively selected another 1:1 matched series of SRSTI patients (non-traction group) undergoing non-traction PVCR from January 2005 to September 2017. We selected 1:1 matched patients meeting all of the following criteria: same sex; age differences within ±5 years; same apical vertebra of the main curves (proximal thoracic, main thoracic, thoracolumbar, and lumbar); the same number of lateral curves; and Cobb angles within ± 20°.

### 2.1. Skull-femoral traction protocol

The patient was placed in the supine position and under local anesthesia. Gardner-Wells tongs were placed below the cranial equator. For both femoral tractions, a 4-mm Steinman pin was inserted 3 cm above the superior border of the patella. Skull-femoral traction started with a weight of 9 kg (skull 3 kg; each femur 3 kg) and gradually decreased at 3 kg (skull 1 kg; each femur 1 kg) per day unless the patient exhibited significant intolerance. The maximum load was equal to 50% of the patient’s body weight. To counteract the tendency for traction weights, the patient was placed by raising the foot of the bed. During traction, the patient was given a noninvasive ventilator BiPAP treatment. Balloon-blowing exercises were performed to improve respiratory function. Muscle strengthening exercises were performed to slow down bone loss and muscle atrophy. The patient used specific pressure-relieving mattresses to prevent pressure ulcers or bed sores. Pin care was performed as needed. Standard full-spine X-rays were taken every week (supine full-spine anteroposterior X-rays).^[13]^

**Figure 2.**
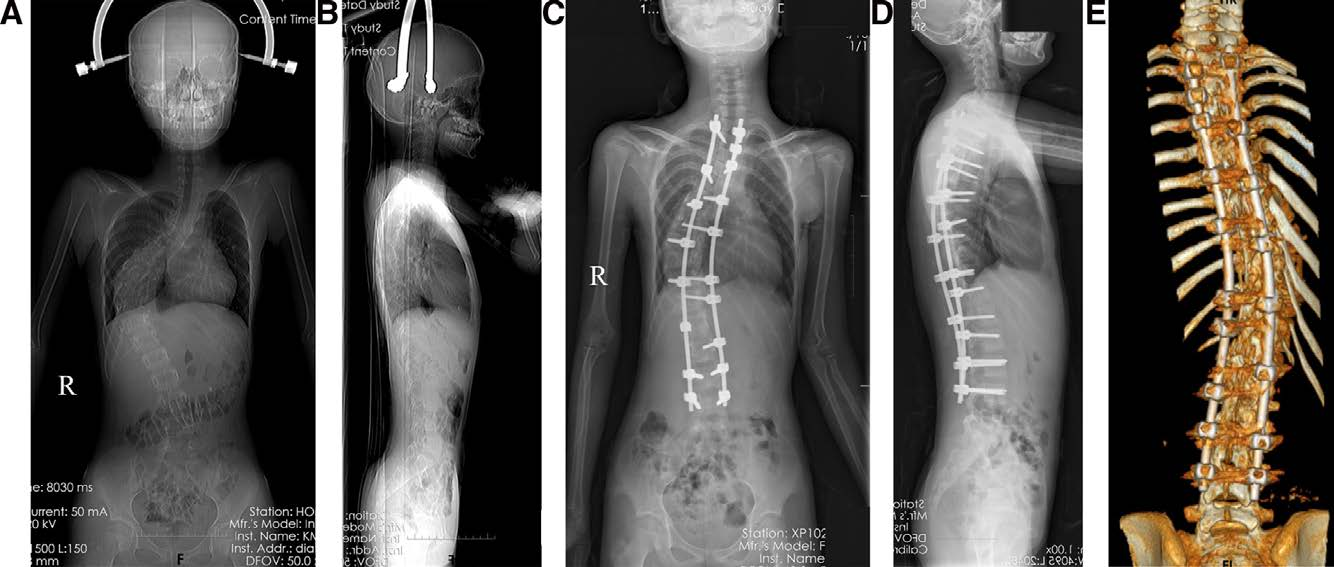
(A) After 15-day skull-femoral traction, an AP X-ray shows an improved Cobb angel (65°), RSH (1.1 cm), and C7-central sacral vertical line (CSVL; 2.6 cm). (B) A lateral X-ray shows an improved Cobb angle (42°) and SVA (0 cm). (C) An AP X-ray taken immediately after surgery, deformity correction, T2-L4 fixation with pedicle screws and rods, and spinal fusion with bone grafts, and thoracoplasty on the convex side. It also shows a Cobb angle of the coronal main curve of 49°, RSH of 1 cm, and C7-CSVL of 2.8 cm. (D) On a lateral X-ray, the Cobb angle is 31°, and the SVA is 0 cm. (E) Postoperative 3-dimensional CT image reconstruction showing posterior spine and implants. AP = anteroposterior, C7-CSVL = C7-central sacral vertical line, CT = computed tomography, RSH = radiographic shoulder height, SVA = sagittal vertical axis.

We examined the patient 3 times a day on the function of the cranial nerves, movement of upper and lower limbs, intestine and bladder function, traction site infection, eating, sleeping, urinating, and defecating. If the patient could not tolerate traction, we decreased the load from 9 to 3 kg until normal nerve function had recovered. Usually, the major curvature rapidly improved in the initial 2 weeks and gradually improved thereafter (Fig. 2A, B).

### 2.2. PVCR and deformity correction

The operation was performed on a Jackson spine table and under general anesthesia and intubation. The patient was placed in a prone position with good padding. During surgery, skull-femoral traction was maintained with 50% of the preoperative load with intraoperative neurophysiological monitoring. A posterior middle incision was made. The vertebrae were exposed through the posterior approach.^[10]^ Under fluoroscopic guidance, pedicle screws were placed. Grade 1 to 6 osteotomies were performed based on the severity of deformity and preoperative plan. PVCR was performed with temporary rod fixation and protection. A total spondylectomy was performed to remove the entire vertebral body, adjacent discs, and associated posterior elements. Only the spinal cord was preserved. Spine curvature was sequentially corrected with all multiaxial pedicle screws and rods. The vertebral space was slightly decreased to prevent hypertension and ischemia of the spinal cord. A cage and bone grafts were implanted across the space between the cephalad and caudal vertebrae. Satisfactory correction and implant position were confirmed using fluoroscopy. The wound was closed in the usual manner. During surgery, neuroelectrophysiological monitoring helped assess the function of the spinal cord and nerve roots.

### 2.3. Postoperative management

The patient was placed on bed rest for 1 to 2 weeks, followed by walking with a supporting brace for 3 months. The patients were prescribed eating patterns based on the detailed dietary guidelines under daily inspection and supervision. Under the guidance of the accelerated recovery surgical team, the patients received regular pulmonary rehabilitation training every day. We recommended muscle strength training such as leg raising straight sport and swimming.

### 2.4. Outcome evaluation

**Figure 3.**
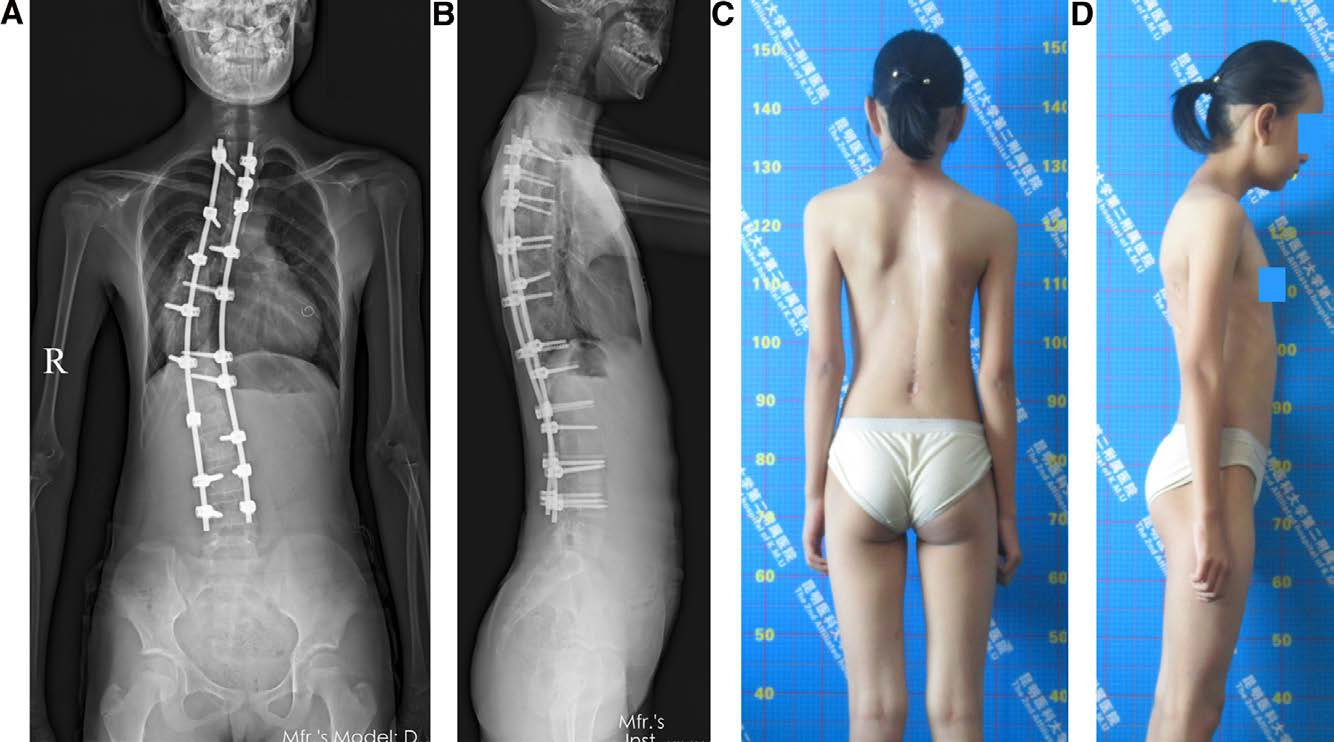
Results 26 months after surgery. (A) An AP X-ray shows a Cobb angle of the coronal main curve of 51°, RSH of 1.5 cm, and C7-CSVL of 1.17 cm. (B) On a lateral X-ray, the Cobb angle is 31°, and the SVA is 1.4 cm. (C) Posterior view. (D) Lateral view. AP = anteroposterior, C7-CSVL = C7-central sacral vertical line, RSH = radiographic shoulder height.

Radiographical parameters were taken at the patient’s admission (Fig. 1C–E), after traction (Fig. 2A, B), immediately after surgery (Fig. 2C, D), and at the last follow-up visit (Fig. 2E, F). In traction, X-rays were taken with the patient in supine position. We assessed Cobb angles on the coronal and sagittal planes, radiographic shoulder height (RSH), C7-center sacral vertical line, and sagittal vertical axis (SVA).^[3]^ The types of spinal curvature, angle, and apex location were assessed based on the Classification for Severe Spinal Deformity-Based X-ray Feature.^[14]^ We used the 36-item short-form (SF-36) to survey the patient’s health status.^[15]^ We used the Scoliosis Research Society-22 (SRS-22) Questionnaire Score, which comprises 22 questions, to assess the domains of function, pain, and mental health.^[16–19]^ At the final follow-up, the patients were invited to the hospital for assessments (Fig. 3A–D). A senior spine surgeon who did not attend the treatments made all assessments.

### 2.5. Statistical analysis

Quantitative variables were described as mean and standard deviation for symmetric distribution or median and interquartile range for asymmetric distribution. Data were compared with the Wilcoxon signed-rank test (to test whether there is a significant difference between 2 population means) and single factor paired logistic regression (to reduce several variables by combining them into a single factor). The significance level was α = 0.05. Differences were considered statistically significant at *P* < .05. The data were analyzed with the Statistical Package for Social Sciences 26.0 (SPSS 26.0, Inc., Chicago).

## 3. Results

**Figure 4.**
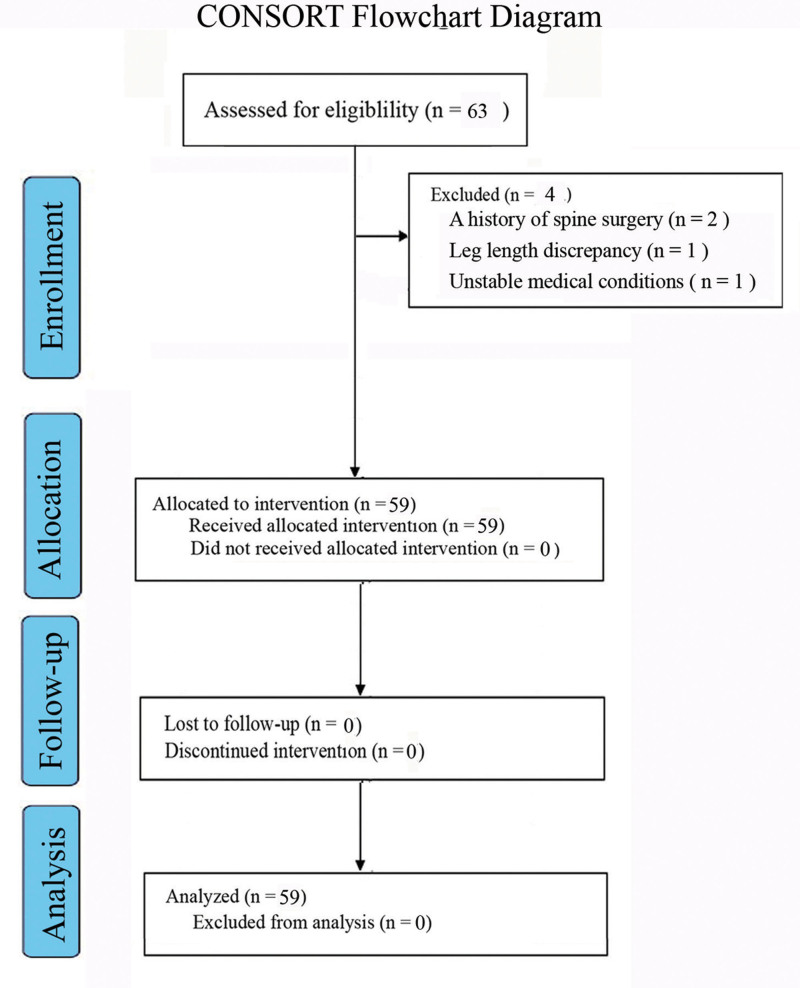
CONSORT flowchart diagram for the traction group.

In the traction group, 63 patients were selected, and 59 patients were finally analyzed (Fig. 4). The age of the traction group and non-traction group were 21.1 ± 8.2 years (range, 11–42 years) and 21.4 ± 10.3 years (range, 10–44 years), respectively (*P* = .287; Table 1). There were 27 male patients and 32 female patients in each group. The apical vertebrae were the same in 2 groups (proximal thoracic, n = 5; main thoracic, n = 37; thoracolumbar, n = 13; and lumbar, n = 4). Total SRS-22 Score assessed at admission was 1.9 (range, 1.8–1.95) vs 1.95 (range, 1.85–2; *P* = .243; Table 2).

**Table 1:** Preoperative clinical characteristics and radiological parameters for 118 patients.

	Traction	Non-traction	*P* value	OR (95% CI)
	(n = 59)	(n = 59)		
Age (yr, mean)	21.1 ± 8.2	21.4 ± 10.3	.287	0.901 (0.674, 1.121)
Sex (male:female)	27:32	27:32		
Height (m)	1.47 ± 0.18	1.51 ± 0.57	.23	0.842 (0.563, 1.325)
Weight (kg)	41.39 ± 2.36	44.28 ± 3.21	.84	0.931 (0.446, 3.92)
BMI (kg/m^2^)	19.54 ± 3.35	21.61 ± 4.75	.11	1.379 (0.965, 1.35)
Prior treatment	None	None		
Apical vertebra			.000	0.000
Proximal thoracic	5	5		
Main thoracic	37	37		
Thoracolumbar	13	13		
Lumbar	4	4		
Number of scoliosis (n)			.000	0.000
2	27	27		
3	32	32		
Cobb angle in coronal plane (°)	113 ± 17	108 ± 19	.131	1.131 (0.901, 1.04)
Cobb angle in sagittal plane (°)	81 ± 32	83 ± 31	.965	1.025 (0.934, 1.210)
Radiographic shoulder height (cm)	2.2 ± 1.1	2.4 ± 1.3	.521	0.876 (0.541, 1.312)
C7 plumb line-center sacral vertical line (cm)	3.2 ± 2.2	3.1 ± 2.3	.72	1.23 (0.745, 1.390)
Sagittal vertical axis (cm)	2.8 ± 1.8	3.4 ± 2.3	.234	0.761 (0.452, 1.428)
Shoulder balance (n, yes: no)	22: 37	25: 34	.714	1.089 (0.354, 3.321)
Coronal balance (n, yes: no)	23:36	24: 35	.97	1 (0.075, 15.891)
Sagittal balance (n, yes: no)	28: 31	31: 28	.747	0.8 (0.232, 2.870)
Traction duration (d, mean, range)	24 (14–39)			
Follow-up (month)	34.9 ± 7.3	31.1 ± 6.6	.714	1.021 (0.915, 1.035)

Data are analyzed with single factor paired logistic regression. On X-rays, the Cobb angle in the coronal plane is the angle between a line parallel to the top of the uppermost vertebra and a line parallel to the bottom of the lowest vertebra in sagittal plane; the Cobb angle in the sagittal plane is the angle formed by the intersection of perpendiculars to the parallel lines drawn to the upper and lower plates of the curve-limiting vertebrae; radiographic shoulder height is the height difference of soft tissue shadows directly superior to acromioclavicular. Sagittal vertical axis refers to the distance between a plumb line dropped from the body of the C7 vertebra to the posterosuperior corner of S1.

BMI = body mass index, CI = confidence interval, OR = odds ratio.

**Table 2:** Assessments at admission.

	Traction	Non-traction		
	(n = 59)	(n = 59)	*P* value	*Z*
SF-36 (%, mean, range)				
Physical functioning	35 (30–40)	37 (25–40)	.133	0.270
Role limitations due to physical health	31 (25–50)	33 (25–50)	.470	−0.220
Role limitations due to emotional problems	41 (21–64)	44 (42–64)	.563	1.609
Emotional well-being	64 (60–84)	54 (64–88)	.201	−2.132
Social functioning	40 (25–50)	38 (12.5–37.5)	.287	−1.70
Pain	61 (42–74)	54 (64–80)	.400	0.336
General health	22 (10–52)	26 (15–57)	.107	0.296
Health change	57 (25–77)	69 (50–75)	.389	2.675
Total SRS-22 Score (mean, range)	1.9 (1.8–1.95)	1.95 (1.85–2)	.243	1.019

Data are compared with a Wilcoxon signed-rank test.

SF-36 = the Short-Form Health Survey, SRS-22 = Scoliosis Research Society-22.

Of the traction group, the duration of skull-femoral traction was 24 days (range, 14–39 days). Radiographic parameters improved after traction (Table 3). The spinal curvature included types SS (n = 6), KS (n = 8), AD (n = 12), LC (n = 22), and DC (n = 11). The curvature angles included types A (n = 23), B (n = 25), C (n = 3), and D (n = 8). The apex levels ranged from T4 to L3, including T4 (n = 5), T5 (n = 3), T6 (n = 3), T7 (n = 6), T8 (n = 14), T9 (n = 19), T12 (n = 3), L2 (n = 3), and L3 (n = 3). Fixation extent included T1-L4 (n = 3), T1-S1 (n = 6), T2-L2 (n = 3), T2-L4 (n = 16), T2-L5 (n = 11), T2-S1 (n = 8), T3-L3 (n = 3), T4-L4 (n = 3), T4-L51 (n = 3), and T6-L4 (n = 3). The pre- and post-traction Cobb angles in the coronal plane were 113°±17° and 77°±21°, respectively (*P* < .001). The Cobb angles in the sagittal plane were 81°±32° and 62°±23°, respectively (*P* = .005). The RSH was 2.2 ± 1.1 cm and 1.3 ± 0.8 cm, respectively (*P* = .014). The C7-CSVL was 3.2 ± 2.2 and 1.4 ± 0.9, respectively (*P* = .013). The SVA of the traction and non-traction groups were 2.8 ± 1.8 cm and 1.4 ± 1.1 cm, respectively (*P* = .19; Table 4). The osteotomies included grade 1 (n = 26), 2 (n = 11), and 5 (n = 22). The operative time was 521 ± 101 minutes, and the amount of intraoperative blood loss was 987 ± 446 mL. Intraoperative neurophysiological monitoring alert occurred in 2 patients. Postoperative complications were transient urinary retention (n = 3), atelectasis (n = 5), superficial wound infection (n = 4), and digestive system dysfunction (n = 4). Radiographic parameters improved after traction (Tables 5 and 6).

**Table 3:** Comparing the radiographic parameters collected before and after skull-femoral in the traction group (n = 59).

	Before traction*	After traction	*P* value	*Z*
Cobb angle in coronal plane (°)	113 ± 17	77 ± 21	<.001	−3.671
Cobb angle in sagittal plane (°)	81 ± 32	62 ± 23	.005	−2.763
Radiographic shoulder height (cm)	2.2 ± 1.1	1.3 ± 0.8	.014	−2.416
C7 plumb line-center sacral vertical line (cm)	3.2 ± 2.2	1.4 ± 0.9	.013	−2.468
Sagittal vertical axis (cm)	2.8 ± 1.8	1.4 ± 1.1	.019	−2.341

Data are compared with a Wilcoxon signed-rank test.

* Before traction data refers to data recorded at admission.

**Table 4:** Surgical details for 118 patients.

	Traction	Non-traction	*P* value	OR (95% CI)
	(n = 59)	(n = 59)		
Osteotomy grade (n)			.034	0.621 (0.418, 0.894)
1	26	14		
2	11	0		
3	0	0		
4	0	0		
5	22	37		
6	0	8		
Posterior vertebral column resection (n)	22	45	.041	0.2 (0.039, 0.831)
ANRV (n)	0.4 ± 0.3	1.1 ± 0.6	.025	0.178 (0.048, 0.838)
Average number of fused vertebrae (n)	13 ± 1.6	14.8 ± 2.7	.781	1.016 (0.722, 1.320)
Operative time (minute)	521 ± 101	679 ± 443	.040	0.921 (0.911, 1.023)
INMA (n)	2	4	.434	0.825 (0.749, 1.004)
Intraoperative blood loss (mL, mean)	987 ± 446	5961 ± 3214	.047	0.971 (0.891, 1.023)
Complication (n)	16	14	.897	1.41 (0.289, 4.417)
Transient urinary retention	3	2		
Atelectasis	5	7		
Superficial wound infection	4	3		
Digestive system dysfunction	4	2		
Hospital stay (d, mean, range)	52.1 ± 11.9	41.3 ± 17.8	.235	1.109 (0.892, 1.307)
Cost (US$, mean, range)	15,132 (11,209–23,214)	14,516 (9867–17,465)	.268	0.144

Data are analyzed with single factor paired logistic regression.

ANRV = average number of resected vertebrae, INMA = intraoperative neurophysiological monitoring alert.

**Table 5:** Comparison of radiographic parameters within groups at admission and after surgery.

	At admission	After surgery*	*P* value	*Z*
Traction group				
Cobb angle in coronal plane (°)	113 ± 17	49 ± 10	<.001	−4.023
Cobb angle in sagittal plane (°)	81 ± 32	40 ± 12	<.001	−3.645
Radiographic shoulder height (cm)	2.2 ± 1.1	1.2 ± 0.5	.001	−3.307
C7 plumb line-center sacral vertical line (cm)	3.2 ± 2.2	1.8 ± 1.3	.043	−1.823
Sagittal vertical axis (cm)	2.8 ± 1.8	1.4 ± 0.9	.050	−1.886
Non-traction				
Cobb angle in coronal plane (°)	108 ± 19	48 ± 15	<.001	−4.329
Cobb angle in sagittal plane (°)	83 ± 31	39 ± 12	<.001	−3.485
Radiographic shoulder height (cm)	2.4 ± 1.3	1.8 ± 0.5	.020	−2.332
C7 plumb line-center sacral vertical line (cm)	3.1 ± 2.3	2.0 ± 0.8	.028	−3.129
Sagittal vertical axis (cm)	3.4 ± 2.3	1.9 ± 0.8	.026	−2.884

Data are compared with a Wilcoxon signed-rank test.

* Refers to 0 to 7 days after surgery.

**Table 6:** Comparison of radiographic parameters within and between groups at the final follow-up.

	Traction	Non-traction		
	(n = 59)	(n = 59)	*P* value	OR (95% CI)
Cobb angle in coronal plane (°)				
Immediately after surgery	49 ± 10	48 ± 15	.922	0.965 (0.747, 1.235)
Final follow-up	50 ± 12	49 ± 13	.907	1.212 (0.912, 1.112)
*P* value	.112	.089		
*Z*	−1.634	−1.713		
Cobb angle in sagittal plane (°)				
Immediately after surgery	40 ± 12	39 ± 12	.603	1.132 (0.901, 1.328)
Final follow-up	42 ± 11	40 ± 12	0.904	0.928 (0.938, 1.246)
*P* value	.048	.023		
*Z*	−1.871	−2.312		
Radiographic shoulder height (cm)				
Immediately after surgery	1.2 ± 0.5	1.8 ± 0.5	0.987	0.21 (0.039, 1.212)
Final follow-up	1.2 ± 0.5	1.4 ± 0.8	0.945	0.14 (0.020, 1.331)
*P* value	.321	.456		
*Z*	−1.021	−0.624		
C7 plumb line-center sacral vertical line (cm)				
Immediately after surgery	1.8 ± 1.3	2.0 ± 0.8	0.480	0.786 (0.401, 1.426)
Final follow-up	4.2 ± 1.6	1.8 ± 0.7	0.229	1.874 (0.623, 5.011)
*P* value	.910	.079		
*Z*	−0.845	−1.634		
Sagittal vertical axis (cm)				
Immediately after surgery	1.4 ± 0.9	1.9 ± 0.8	0.165	0.522 (0.167, 1.237)
Final follow-up	1.7 ± 0.6	1.9 ± 0.7	0.313	1.56 (0.643, 4.507)
*P* value	.328	.877		
*Z*	−0.827	−0.166		

Data are compared with a paired *t*-test and Wilcoxon signed-rank test.

CI = confidence interval, OR = odds ratio.

Of the non-traction group, spinal curvature included types SS (n = 6), KS (n = 9), AD (n = 11), LC (n = 25), and DC (n = 8). The curvature angles included A (n = 26), B (n = 22), C (n = 5), and D (n = 6). The apex levels ranged from T2 to L2, including T2 (n = 3), T6 (n = 3), T8 (n = 16), T9 (n = 31), T12 (n = 3), and L2 (n = 3). Fixation extent included C7-T11 (n = 3), T1-L3 (n = 3), T1-L4 (n = 3), T1-L5 (n = 6), T1-S1 (n = 10), T2-L2 (n = 5), T3-L3 (n = 3), T2-L4 (n = 5), T2-L5 (n = 3), T2-S1 (n = 3), T3-L3 (n = 3), T3-S1 (n = 3), T4-L4 (n = 3), T1-S1 (n = 3), and T8-L5 (n = 3). Osteotomies were grade 1 (n = 14), 5 (n = 37), and 6 (n = 8) osteotomies, and the operative duration was 679 ± 443 minutes. The amount of intraoperative blood loss was 5961 ± 3214 mL. Intraoperative neurophysiological monitoring alert occurred in 5 patients. Postoperative complications included transient urinary retention (n = 2), atelectasis (n = 7), superficial wound infection (n = 3), and ileus (n = 2). There were significant between-group differences in osteotomy grade (*P* = .034; OR, 0.621), number of vertebral column resection (*P* = .041; OR, 0.2), each patient’s number of resected vertebrae of (*P* = .025; OR, 0.178), operative time (*P* = .040; OR, 0.921), and intraoperative blood loss (*P* = .047; OR, 0.971).

**Figure 5.**
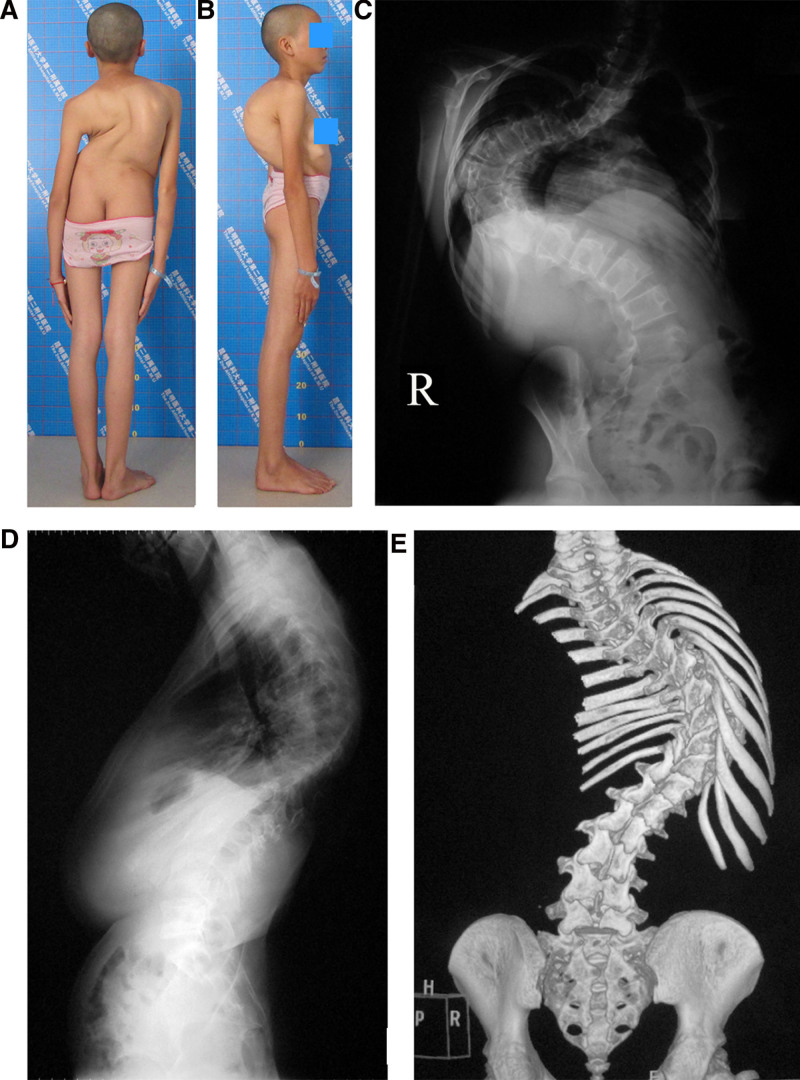
A 12-year-old female patient suffers from severe rigid scoliosis with trunk imbalance. (A) Preoperative deformity in the posterior view. (B) Lateral view. (C) A preoperative anteroposterior (AP) X-ray shows a Cobb angle of 132°, radiographic shoulder height (RSH) of 4.1 cm, C7-central sacral vertical line (C7-CSVL) of 3.2 cm. (D) On a lateral X-ray, the Cobb angle is 65°, and the sagittal vertical axis (SVA) is 4.0 cm. (E) Preoperative 3-dimensional CT image reconstruction showing a posterior view of the spinal deformity. AP = anteroposterior, C7-CSVL = C7-central sacral vertical line, CT = computed tomography, RSH = radiographic shoulder height, SVA = sagittal vertical axis.

**Figure 6.**
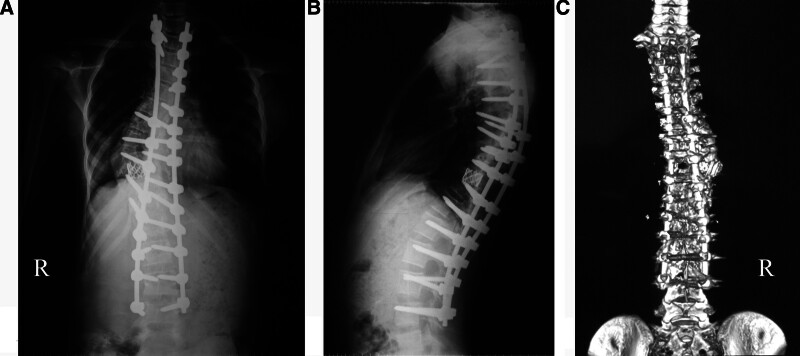
Posterior vertebral column resection (PVCR) at T10 level to correct the deformity, spinal shortening, deformity correction, T2-L4 fixation with pedicle screws and rods, and spinal fusion with titanium mesh and bone grafts. (A) A postoperative AP X-ray shows a Cobb angle of the coronal main curve of 22°, RSH of 0 cm, and C7-CSVL of 1.5 cm. (B) On a lateral X-ray, the Cobb angle is 34°, and the SVA is 2.4 cm. (C) Postoperative 3-dimensional CT image reconstruction showing posterior spine and implants. C7-CSVL = C7-central sacral vertical line, CT = computed tomography, PVCR = posterior vertebral column resection, RSH = radiographic shoulder height, SVA = sagittal vertical axis.

**Figure 7.**
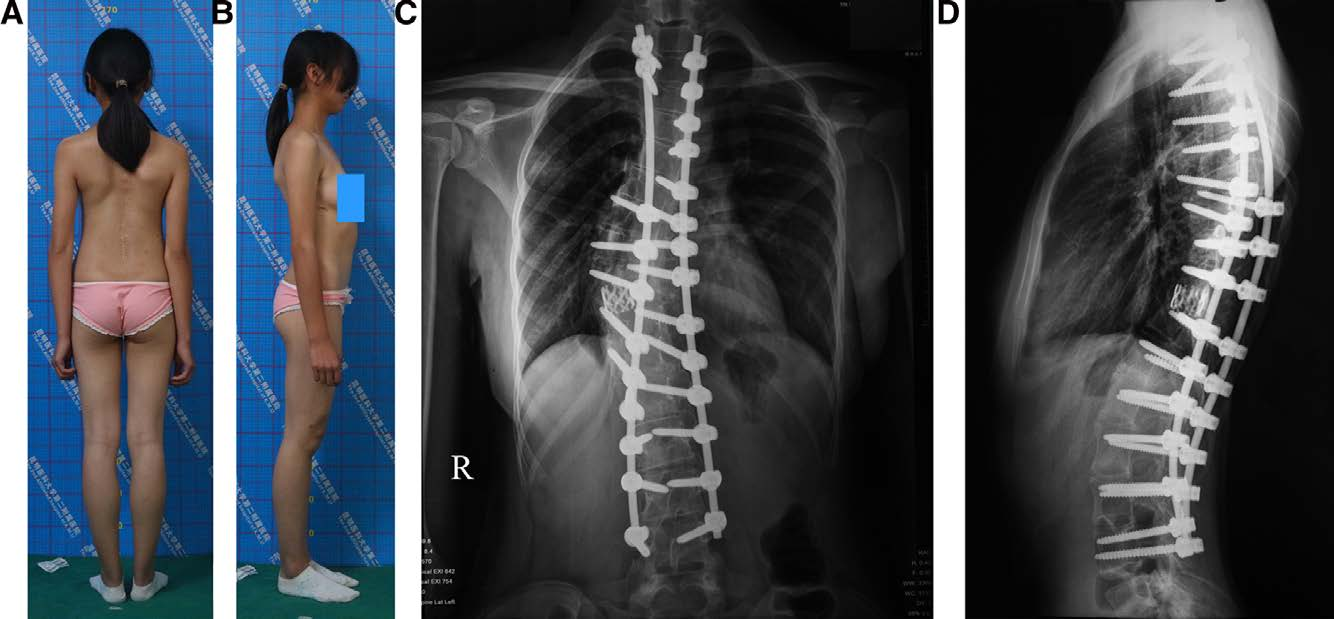
Forty-eight months after surgery. (A) Posterior view. (B) Lateral view. (C) An AP X-ray shows a Cobb angle of the coronal main curve of 14°, RSH of 0 cm, and C7-CSVL of 0.3 cm. (D) A lateral X-ray shows the Cobb angle 25° and the SVA 1 cm. AP = anteroposterior, C7-CSVL = C7-central sacral vertical line, RSH = radiographic shoulder height, SVA = sagittal vertical axis.

Follow-ups of the 2 groups lasted for 34.9 ± 7.3 (range, 26–49) months and 31.1 ± 6.6 (range, 25–51) months, respectively (*P* = .714; OR, 1.021). For the 36-Item Short-Form Survey Instrument, the physical functioning and general health were 96 (range, 84–100) and 84 (range, 67–80) vs 93 (range, 86–100) and 80 (range, 69–80), respectively (*P* = .141 and 0.220). Total SRS-22 Score were 4.7 (range, 3.8–5) and 4.5 (range, 3.5–5), respectively (*P* = .298; Table 7). Figures 5–7 show another SRSTI patient.

**Table 7:** Functional assessments at the final follow-up.

	Traction	Non-traction		
	(n = 59)	(n = 59)	*P* value	*Z*
SF-36 (%, mean, range)				
Physical functioning	96 (84–100)	93 (86–100)	.141	0.303
Role limitations due to physical health	94 (76–100)	96 (86–100)	.532	−0.420
Role limitations due to emotional problems	91 (68–100)	93 (82–100)	.781	1.409
Emotional well-being	97 (87–100)	92 (81–100)	.122	−1.320
Social functioning	86 (74–100)	82 (76–100)	.246	−1.31
Pain	46 (37–50)	44 (37–50)	.501	1.01
General health	84 (67–80)	80 (69–80)	.220	0.245
Health change	91 (76–100)	89 (74–100)	.334	1.966
Total SRS-22 Score (mean, range)	4.7 (3.8–5)	4.5 (3.3–5)	.298	1.299

Data are compared with the Wilcoxon signed-rank test.

SF-36 = the Short-Form Health Survey, SRS-22 = Scoliosis Research Society-22.

## 4. Discussion

In the treatment of SRSTI, preoperative skull-femoral traction improves preoperative curve flexibility of the spine. This improvement decreases difficulties of PVCR and thereby decreases osteotomy grade, vertebral column resection, number of 3-column vertebrae resection, the amount of intraoperative blood loss, and the risks of iatrogenic injuries. However, both traction and non-traction PVCR achieve similar spine correction and functional outcomes with similar complication and modality rates.

A shoulder height difference >2 cm may significantly affect the patient’s satisfaction and social psychology. SRSTI is often combined with both coronal and sagittal plane imbalance, resulting in pelvic, hip, and knee compensation. The outside-spine compensation (posterior pelvic tilt, hip flexion, knee flexion, etc) often occurred in the sagittal plane but the coronal plane (Fig. 1A, B). Trunk imbalance is usually confirmed by measuring the horizontal distance between the C7 plumb line and central sacral vertical line on full-length spine standing anteroposterior X-rays.^[1]^ Coronal imbalance is confirmed when a C7 plumb line-center sacral vertical line (C7PL-CSVL) > 2 cm. Sagittal imbalance is confirmed when a SVA > 2.5 cm. Shoulder imbalance is confirmed when RSH > 2 cm.^[3]^ We discuss the following issues on preoperative traction and PVCR.

### 4.1. Intraoperative blood loss

Suk et al^[20]^ first performed PVCR for severe spinal deformity in 2002. The technique has been used worldwide thereafter, but difficulties and blood loss increase with the severity of the preoperative deformity, osteotomy grade, and multi-vertebral resection. Ligating and cauterizing segmental arteries can reduce intraoperative bleeding but compromise the blood supply to the spinal cord. In an animal study, Nambu et al^[21]^ found that blood perfusion decreased to 70% after ligating 1 segmental artery.

### 4.2. Spinal traction

Seller et al^[22]^ treated 25 patients with severe neuromuscular spine deformity. Eight patients had preoperative Halo-traction, and 17 patients underwent direct operative correction. They achieved 57% correction in traction patients, and 61% correction in non-traction patients. Sponseller et al^[23]^ performed a multicenter study involving 53 patients with severe scoliosis or kyphosis. Thirty patients were treated with halo-gravity traction, and 23 were treated without traction. There was no difference in main coronal curve correction (62% vs 59%), operative time, blood loss, or complication rate (27% vs 52%). However, the non-traction patients underwent more vertebral column resection (30% vs 3%). The traction patients had a longer of hospital stay (36 vs 14 days). Bogunovic et al^[24]^ performed preoperative halo-gravity traction in 33 patients with severe scoliosis for 70 days. The traction weight applied was 39% of the total body weight. They achieved 35% correction without serious complications.

The main drawback of halo-gravity traction was the prolonged length of hospital stay. Kato et al^[14]^ treated 121 patients with idiopathic scoliosis. They found that intraoperative skull-femoral traction with high traction weight (≥35% of body weight) could achieve a more significant correction but with a 3-time increased risk of motor-evoked potential change, compared to low traction weight (<35% of body weight). Qiao et al^[16]^ treated 63 severe scoliosis patients with 3-stage correction (stage I, posterior release and screw placement; stage II, traction for 22 days; and stage III, posterior instrumentation). The skull-femoral traction weight was 28.4 kg, equal to 57% of the patient’s body weight. The postoperative correction rate was 55% for scoliosis and 51% for kyphosis. However, traction-related complications often occurred after 3 weeks with incidences ranging from 16% to 28%, including pin site infection, pin loosening, deep vein thrombosis, and gastrointestinal and neurological symptoms. Therefore, short-term (<14 days) skull-femoral traction generates greater traction forces with fewer complications, and those complications are often managed easily.

### 4.3. Indications

The ideal candidates for preoperative skull-femoral traction and PVCR are SRSTI patients with a Cobb angle of the main curve >90° and flexibility <20%; imbalance patient, especially coronal imbalance that can be confirmed easily after traction; the need for improvement of preoperative spinal curvature to mitigate the neurological risks; the need for slow correction to decrease the risk of spinal cord injuries in severe deformities; and a surgical plan based on the surgeons’ experience and preference. Contraindications are the presence of intra- or extradural growths and medullar canal stenosis, with or without neurological deficits.

### 4.4. Limitations

This study has limitations. First, a small number of patients and a short follow-up period may affect the reliability of the outcomes. Second, measurement errors may occur due to nonstandard bedside X-rays. Third, the psychological status of the patients should be assessed using reliable questionnaires. Fourth, a retrospective, unrandomized, and unblinding design produces a selection bias in the form of differences in a broader population, which has a critical impact on the study outcomes. A further study is needed to explore the characteristics of surgical treatment of patients with different etiology.

## 5. Conclusions

In conclusion, for the treatment of SRSTI, preoperative skull-femoral traction enhances spinal curve flexibility, leading to a reduction in osteotomy grade, PVCR, the average number of 3-column vertebrae resections, operative time, and intraoperative blood loss. However, both traction and non-traction techniques achieve similar spine correction and functional outcomes with similar complications and modalities.

## Author contributions

**Conceptualization:** Yingliang Liu.

**Data curation:** Changlei Xu, Xiaobing Tian.

**Formal analysis:** Changlei Xu.

**Funding acquisition:** Yingsong Wang.

**Investigation:** Changlei Xu.

**Methodology:** Yingliang Liu.

**Project administration:** Yingliang Liu.

**Resources:** Jingming Xie.

**Software:** Yingliang Liu.

**Supervision:** Yingsong Wang.

**Validation:** Ni Bi.

**Visualization:** Jie Xiao.

**Writing – original draft:** Yingliang Liu.

**Writing – review & editing:** Zhi Zhao.
